# An Interplay between Oxidative Stress (Lactate Dehydrogenase) and Inflammation (Anisocytosis) Mediates COVID-19 Severity Defined by Routine Clinical Markers

**DOI:** 10.3390/antiox12020234

**Published:** 2023-01-20

**Authors:** Marta Alonso-Bernáldez, Amanda Cuevas-Sierra, Víctor Micó, Andrea Higuera-Gómez, Omar Ramos-Lopez, Lidia Daimiel, Alberto Dávalos, María Martínez-Urbistondo, Víctor Moreno-Torres, Ana Ramirez de Molina, Juan Antonio Vargas, J. Alfredo Martinez

**Affiliations:** 1 Precision Nutrition and Cardiometabolic Health, IMDEA Food Institute, CEI UAM+CSIC, 28049 Madrid, Spain; 2 Centro de Investigación Biomédica en Red Fisiopatología de la Obesidad y la Nutrición (CIBEROBN), Instituto de Salud Carlos III, 28049 Madrid, Spain; 3 Medicine and Psychology School, Autonomous University of Baja California, Tijuana 22390, Mexico; 4 Nutritional Control of the Epigenome Group, IMDEA Food Institute, CEI UAM+CSIC, 28049 Madrid, Spain; 5 Departamento de Ciencias Farmacéuticas y de la Salud, Facultad de Farmacia, Universidad San Pablo-CEU, CEU Universities, Urbanización Montepríncipe, 28660 Boadilla del Monte, Spain; 6 Epigenetics of Lipid Metabolism Group, IMDEA Food Institute, CEI UAM+CSIC, 28049 Madrid, Spain; 7 Puerta de Hierro Research Institute, University Hospital, Majadahonda, 28222 Madrid, Spain; 8 UNIR Health Sciences School Medical Center, Pozuelo de Alarcón, 28040 Madrid, Spain; 9 Molecular Oncology and Nutritional Genomics of Cancer Group, IMDEA Food Institute, CEI UAM+CSIC, 28049 Madrid, Spain

**Keywords:** oxidative stress, LDH, anisocytosis, interaction, COVID-19

## Abstract

Viral infections activate the innate immune response and the secretion of inflammatory cytokines. They also alter oxidative stress markers, which potentially can have an involvement in the pathogenesis of the disease. The aim of this research was to study the role of the oxidative stress process assessed through lactate dehydrogenase (LDH) on the severity of COVID-19 measured by oxygen saturation (SaO_2_) and the putative interaction with inflammation. The investigation enrolled 1808 patients (mean age of 68 and 60% male) with COVID-19 from the HM Hospitals database. To explore interactions, a regression model and mediation analyses were performed. The patients with lower SaO_2_ presented lymphopenia and higher values of neutrophils-to-lymphocytes ratio and on the anisocytosis coefficient. The regression model showed an interaction between LDH and anisocytosis, suggesting that high levels of LDH (>544 U/L) and an anisocytosis coefficient higher than 10% can impact SaO_2_ in COVID-19 patients. Moreover, analysis revealed that LDH mediated 41% (*p* value = 0.001) of the effect of anisocytosis on SaO_2_ in this cohort. This investigation revealed that the oxidative stress marker LDH and the interaction with anisocytosis have an important role in the severity of COVID-19 infection and should be considered for the management and treatment of the oxidative phenomena concerning this within a precision medicine strategy.

## 1. Introduction

Since the COVID-19 pandemic started, infection by the SARS-CoV-2 virus has caused more than 6 million deaths and 600 million cases worldwide (Johns Hopkins University, CSSE, accessed on 1 November 2022) [1]. This new coronavirus has shown proneness to mutate, engendering newer variants with increased transmissibility, variable pathogenesis, and global spread [2]. The fast development of vaccines against SARS-CoV-2 has helped to manage this viral disease, which not only causes respiratory complications such as hypoxemia but also liver and gastrointestinal tract-related manifestations and multisystemic symptoms [3]. However, research on the physiopathology of this virus, as well as the analysis of risk factors associated with severe COVID-19 infection, remain key to design effective forms of prevention, stratification, and management [4]. Economic efforts should be directed toward finding easy and fast strategies to identify the potential cases at most risk and advance ahead of the progression of the disease, since the associated costs in healthcare resources derived from the management of severe COVID-19 and associated metabolic determinants, such as the presence of abnormal or excessive fat, cost billions to governments [5]. Moreover, changes in insulin resistance, coagulation processes, and apoptosis have been reported in these patients, pointing out the role of metabolic syndrome in COVID-19 [6,7,8]. These conditions increase the mortality associated with this viral infection by decreasing blood oxygen saturation (SaO_2_), a key clinical parameter in this disease [9,10], and altering the redox status [11].

In the COVID-19 clinical framework, the relationships between oxidative stress and inflammation are key to understand this viral pathology. It has been described that the entry of SARS-CoV-2 into the cells through the angiotensin-converting enzyme 2 (ACE2) receptor decreases virus bioavailability, which in turn influences the production of reactive oxygen species (ROS) through angiotensin II regulation [12]. Whereas a moderate increase in the production of ROS is a physiological reaction that helps the organism to fight the infection, excessive levels of ROS have the opposite effect and can damage tissue, impair immunological response, and trigger inflammation processes [11,13]. In addition, the inflammatory response associated with a viral infection triggers the release of cytokines, stimulating the production of the master regulator of inflammation, the transcription factor NF-κB [14]. Furthermore, in COVID-19 patients, this release of proinflammatory cytokines is massive and threatening, causing this condition to be a crucial strategy for treatment [15]. Altogether, this vicious circle affects immune function, decreasing the concentrations of neutrophils and lymphocytes [16] and exacerbating the outcome along with decreased SaO_2_ [17].

In this context, previous investigations have revealed that amino acids, matrix endopeptidases, and antioxidant enzymes levels in plasma, three markers linked to immune response, inflammation, and oxidative stress, respectively, are involved in the development and severity of the disease [18,19,20]. A recent systematic review depicts how an increase in oxidative stress can severely affect COVID-19 outcomes by impairing immunocompetence reflected by neutrophils and lymphocytes counts [21,22], thus revealing its importance in this disease’s physiopathology [23]. In this line, COVID-19 patients have underlying hyperinflammation linked to an increased oxidative stress [12,24]. In fact, the exacerbated immunological reaction by the host after SARS-CoV-2 infection is characterized by excessive levels of proinflammatory cytokines, such as interleukins (IL) and C reactive protein (CRP), which constitute important biomarkers for the cytokine storm syndrome characteristic of COVID-19 severity (namely IL-1β, IL-6, IL-8, IL-17, and TNFα that have been associated with severe cases of COVID-19) [16]. These mediators form part of a wide spectrum of laboratory biomarkers that have been associated with severe COVID-19 disease [25]. For instance, an altered red blood cell (RBC) distribution width (RDW), termed anisocytosis, has been associated with COVID-19 mortality as an inflammatory determinant [26], as well as an increased lactate dehydrogenase (LDH) concentration, indicating severe tissue injury and inflammation [27,28]. RDW is a measure of the variability of circulating erythrocyte size and is routinely reported as part of a complete blood count, although it is not typically used clinically. Elevated RDW is associated with poor outcomes in a wide variety of disease states (including cardiovascular disease, stroke, and critical illness). In patients with COVID-19, RDW has been shown to be correlated with IL-6 levels, as well as TNF-α, likely due to IL-6 induction of hepcidin expression, while TNF-α has been linked to erythropoietin resistance. Additionally, syndromes of acutely dysregulated inflammation, such as sepsis, are often accompanied by the suppression of erythrocyte maturation [29]. In this line, D-dimer has been depicted as another biomarker of pulmonary function associated with COVID-19 mortality [30]. Several studies have reported an increase in D-dimer and fibrinogen concentrations in the early stages of COVID-19 disease, which can worsen with chronic diseases such as diabetes, cancer, or obesity. Measuring the level of D-dimer from the early stage of the disease can provide useful information for controlling and managing COVID-19 disease [31]. Altogether, these clinical indicators can be normally obtained in a routine blood test and, therefore, are potential candidates for use in COVID-19 management.

The aim of this study was to investigate the interplay between LDH and anisocytosis as potential routine biomarkers of oxidative stress and inflammation, respectively, modulating COVID-19 severity assessed by SaO_2_.

## 2. Materials and Methods

### 2.1. Study Design and Settings

A retrospective study was performed including subjects hospitalized with COVID-19 infection. This cohort was built including male and female adult patients, who were admitted in the emergency service of the HM Hospitales group in the city of Madrid during the first wave (March–May 2020). The data were collected following valid hospital protocols approved by the Institutional Ethics Board and were collected in accordance with the Declaration of Helsinki (code: 20.05.1627-GHM).

### 2.2. Subjects

This cohort, named COVID-DATA-SAFE-LIFES, enrolled a total of 2307 COVID-19 positive patients with a mean age of 68 years. The criteria followed for inclusion in this cohort were the diagnosis of viral respiratory disease by the physician, adulthood, and the admission in the emergency system of HM Hospitales Madrid from March to May 2020. The exclusion criteria were an age less than 18 years and the absence/missing of relevant clinical data. For this investigation, the subjects were retained according to missing data of oxygen saturation, LDH, and anisocytosis coefficient, resulting in a total of 1808 participants.

### 2.3. Data Collection

The database was built according to in-hospital protocol, which was harmonized and curated for further analysis in the R software (version 4.0.3.) (R Core Team, Vienna, Austria). The following variables were collected and used to assess validated protocols: age, sex, days of hospitalization, SaO_2_, leukocytes, lymphocytes, neutrophils, platelets, eosinophils, monocytes, transaminases, anemia according to the WHO definition [32], CRP, LDH, anisocytosis coefficient, D-dimer, prothrombin time, glucose, and urea. Moreover, health complications (obesity, hypertension, type 2 diabetes, cardiovascular, dementia, liver, endocrine, kidney, or gastrointestinal diseases) were collected as covariates. Endocrine disorders encompassed endocrine cancers and hypo- and hyperthyroidism.

As non-invasive methods to assess liver damage, the AST-to-platelet ratio index (APRI) and fibrosis-4 (FIB-4) index were calculated. The APRI was calculated using 100 × [AST (U/L)/PLT (10^9^/L)]. FIB-4 was calculated using the following formula: age (years) × AST [U/L]/(PLT [10^9^/L] × (ALT [U/L])^1/2^) [33]. FIB-4 < 1.45 was considered within the normal range with a negative predictive value of advanced fibrosis of approximately 90% [34].

### 2.4. Statistical Analyses

The results were expressed as numbers of cases and percentages for qualitative variables and the mean and standard error for the quantitative variables. The normality of the analyzed variables was screened using the Shapiro–Wilk test. The groups of comparison were stablished according to the median of SaO_2_, lymphocytes, CRP, neutrophils-to-lymphocytes ratio (NLR), LDH, and anisocytosis coefficient. The median was selected as cut-off criteria in order to obtain a similar number of subjects in each group of comparison. The statistical differences between groups were assessed using a Student’s *t*-test or Mann–Whitney U test depending on the normality distribution of data. The chi-square was performed for the evaluation of qualitative variables. The participants were first classified according to the median of the following COVID-19 severity variables: SO_2_, lymphocytes, and D-dimer levels. To further characterize the cohort, the participants were then stratified by inflammatory (NLR and anisocytosis) and oxidative stress (LDH) markers.

A linear regression was carried out including an interaction term to explain the severity of COVID-19 in this population measured by SaO_2_ as dependent variables. The model was adjusted by sex, age, and the presence of other respiratory diseases. The mediation by LDH in the relationship between anisocytosis and SaO_2_ was assessed using structural equation modeling following the Zhao et al. approach [35].

The results with a *p* value < 0.05 were considered statistically significant. Stata Program Version 12 (StataCorp LLC, College Station, TX, USA) was used for statistical analyses and figure depiction.

## 3. Results

### 3.1. Clinical and Phenotypical Characterization of the Cohort

A total of 1808 patients integrated the final database for this investigation and presented all the variables used in these analyses (1084 were men and 724 women). The general information about this population and data related with biochemical, hematological determinations, and medical history were compared by the groups (Table 1, Table 2 and Table 3). The patients with SaO_2_ lower than 94% were female, older, and presented higher levels of anisocytosis, LDH, D-dimer, AST, ALT, glucose, CRP, and hemoglobin, as well as higher levels of leukocytes, neutrophils, and neutrophils-to-lymphocytes ratio (NLR). Additionally, the subjects with low SaO_2_ showed higher levels of FIB-4 and more cases of diabetes mellitus, cardiovascular diseases, overweight or obesity, endocrine diseases, hypertension, and other respiratory diseases (Table 1).

Similar differences were found when comparing the median values of lymphocytes. The patients with lower values of lymphocytes were male, older, and presented higher levels of anisocytosis, LDH, glucose, and CRP. The hematological variables in subjects with lower levels of lymphocytes showed significant low values of hemoglobin, eosinophils, leukocytes and monocytes, prothrombin time, and platelets (Supplementary Table S1 shows the normal ranges for these clinical parameters). NLR, FIB-4, and APRI were significantly higher in this group of patients. Additionally, the patients with lower lymphocyte levels presented more cases of other respiratory diseases and hypertension (Table 2). Regarding the patients with higher D-dimer values, they showed a significant decrease in SaO_2_ and hemoglobin. In contrast, they had higher values of days of hospitalization, glucose level, LDH, AST, FIB-4, APRI, CRP, platelets, leukocytes, neutrophils, and NLR, as well as more cases of cardiovascular diseases, anemia, kidney diseases, and hypertension (Table 3).

Similar features were found when comparing the median values of NLR, as shown in Table 2. However, the patients with a lower ratio presented significant lower levels of D-dimer, AST, ALT, and creatinine. The comparison of the oxidative stress marker (LDH) in COVID-19 patients revealed that the subjects with LDH values lower than 544 U/L were young male and presented lower levels of D-dimer, AST, ALT, glucose, CRP, and creatinine, as well as lower levels of leukocytes, neutrophils, NLR, and FIB-4 (Table 4).

The data compared using the median of anisocytosis showed that the patients with more than 12.8 presented were older men with significantly high levels of LDH, CRP, creatinine, leukocytes, neutrophils, NLR, and FIB-4, as well as lower levels of prothrombin time, platelets, hemoglobin, and eosinophils and significantly more cases of diabetes, cardiovascular disease, anemia, liver disease, endocrine disease, other respiratory disease, kidney disease, and hypertension (Table 4). Interestingly, all the groups in the comparisons showed significant differences in age, days of hospitalization, anisocytosis, leukocytes, neutrophils, and FIB-4, as well as LDH, CRP, NLR, and the anisocytosis coefficient.

### 3.2. Exploration of the Role of Anisocytosis and LDH in COVID-19 Severity

Regarding the differences found between these groups of comparisons, a linear regression model was performed. The anisocytosis coefficient, CRP, NLR and LDH were considered for the regression model as important predictors of SaO_2_ levels. This model was also adjusted considering age, sex, and the presence of other respiratory diseases different to COVID-19. The interaction between LDH and anisocytosis level was included, resulting in being statistically significant (*p* value = 0.01) (Table 5).

**Figure 1:**
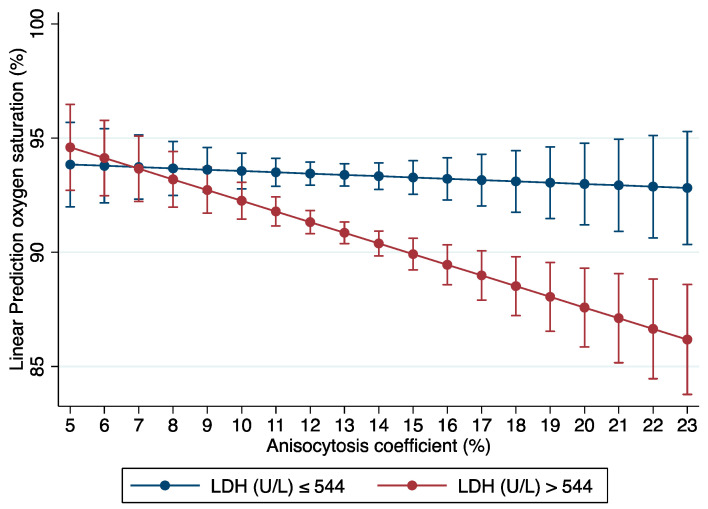
Graph of interaction between LDH level and anisocytosis coefficient as important variables for the prediction of oxygen saturation in COVID-19 patients.

As expected, age, NLR, CRP levels, and the prevalence of other respitaroty diseases were predictors of SaO_2_. An interaction between LDH and the anisocytosis coefficient was found (Figure 1). The LDH levels were categorized according to the median in the population for the graphical presentation. Figure 1 shows predicted SaO_2_ values according to anisocytosis coefficient in low and high LDH participants. In the participants with low LDH levels, a greater anisocytosis coefficient was predicted to not affect SaO_2_. However, in the patients with high LDH levels, greater anisocytosis coefficients were predicted to be associated with lower SaO_2_.

**Figure 2:**
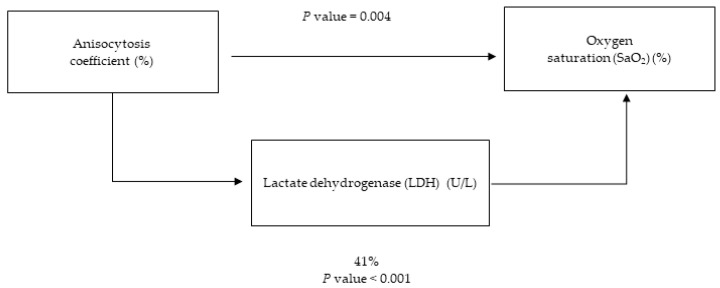
LDH (oxidative marker) level mediation on oxygen saturation (disease severity marker) using anisocytosis coefficient (inflammatory marker) in COVID-19 infected patients.

Since LDH and the anisocytosis coefficient are suggested to be key points for the severity of COVID-19 measured by SaO_2_, a possible mediation by the LDH levels in the relationship between SaO_2_ and anisocytosis was analyzed in this population. The results showed that LDH was mediating 41% (*p* value < 0.001) of the effects of anisocytosis on SaO_2_ (Figure 2).

## 4. Discussion

To further investigate this association, the COVID-19 severity of the present cohort was assessed according to routine markers, such as SaO_2_ (%), lymphocyte count (cell/µL), and D-dimer values (µg/mL); these are three variables that have been associated with fatal outcomes in these patients [36,37]. Age and sex appeared as statistically significant contributors when comparing normal versus altered levels of these determinators. As expected, the days of hospitalization were also significant for the three of them, since the worse the clinical parameters, the more days these patients stay hospitalized. Liver indexes, as well as common haematological variables, such as leukocytes and neutrophils, were also altered in patients with a severe infection by SARS-CoV-2 according to these indicators. Particularly, the FIB-4 index was statistically different in the patients segregated by these severity markers, indicating liver inflammation as a participating factor, which agrees with the previous results [38]. Moreover, the patients with a decreased SaO_2_ also presented increased associated comorbidities, such as diabetes, hypertension, and other metabolic diseases, which agrees with what has been repeatedly reported [36,39].

The patients with increased infection severity showed raised levels of anisocytosis coefficient, D-dimer, and LDH. These variables have been related to severeness and mortality in COVID-19 patients [25]. Firstly, anisocytosis refers to an increased RDW coefficient, revealing an unequal size of RBC, a condition that has been associated with COVID-19 prognosis [40], as well as with a broad spectrum of diseases; it has become a routine and affordable disease indicator of morbidity [41]. RDW has been associated with measures of inflammation, such as CRP, and was linked to the cytokine storm in these patients, highlighting RDW as a potential indicator for high-inflammatory risk in COVID-19 [29]. Moreover, RDW is influenced by oxidative stress, possibly as a recognizing marker of clinical redox status by the increased red cell turnover, contributing to this condition [41]. Regarding LDH, an enzyme involved in the maintenance of the redox state, it catalyzes the oxidation of L-lactate to pyruvate in anaerobic glycolysis by converting NADH to NAD+ [42]. LDH has been pointed out as a biomarker in plasma for lung affectation in COVID-19 patients [30], indicating severe tissue injury and inflammation [27], and has also shown interaction with anti-hypertensive agents as a COVID-19 treatment [43]. It has been shown that LDH levels correlate with oxidative markers, such as plasma peroxides, in COVID-19 patients [44]. Meanwhile, D-dimer is a cleavage product found in plasma as the result of fibrin breakdown and has been related to COVID-19 mortality as a marker of inflammation and thrombosis [25].

To discriminate the morbid impact between inflammatory markers and oxidative stress, the cohort was stratified with high and low levels of anisocytosis, LDH, and NLR. NLR was selected as a marker of inflammation that has been associated with a worse prognosis in COVID-19 patients [25]. The results showed that older patients had increased levels of all these markers compared to the younger, as well as higher hospitalization days. Moreover, the patients with altered levels of NLR, LDH, and anisocytosis presented higher liver indexes, decreased lymphocyte and neutrophil counts, and an increased prevalence of metabolic and related diseases, which agrees with previous findings [36,38,45]. The fact that CRP was increased in patients with altered NLR, LDH, and anisocytosis values demonstrated once again the stormy inflammation associated with SARS-CoV-2 infection, as previously described by several investigations [8,45]. Interestingly, D-dimer was significantly increased in those patients with increased markers of NLR and LDH, but not of anisocytosis. This finding agrees with other authors [26,45], suggesting that D-dimer may be closely related to immunocompetence and that its well-described inflammatory mediation in this disease might not be through RBW but rather by activating the production of cytokines, such as IL-6, as previously reported [46]. Of note, the demonstrated influence of comorbidities, such as diabetes and obesity, on inflammation and oxidative status cannot be ruled out [47]. However, the patients with some of these reported comorbidities constituted a small percentage of the totality of the cohort, with the presence of other respiratory diseases being the most common comorbidity in these patients, as previously reported [48].

A regression model was built using NLR, LDH, anisocytosis coefficient, CRP, and other associated respiratory diseases as clinical elements affecting SaO_2_ in COVID-19 patients, as well as age and sex as covariates. Although LDH and anisocytosis did not affect SaO_2_ independently, the interaction between both was significant, pointing out the relevance of considering them jointly. This finding depicted the influence of LDH on SaO_2_ levels, as the anisocytosis coefficient only affected SaO_2_ if the LDH levels were elevated, showing the importance of oxidative stress when explaining COVID-19 severity. To further explore this interaction, a mediation analysis was performed, which revealed that LDH significantly mediated the effect of anisocytosis on SaO_2_ by 41%. Mediation analyses are a very interesting approach, since they provide insightful information about to what extent a variable affects another and can occur simultaneously with an interaction [49]. This effect can be direct, such as the effect of anisocytosis on SaO_2_ (anisocytosis → SaO_2_), or indirect, the same effect but mediated by LDH (anisocytosis → LDH → SaO_2_). In the latter one, the anisocytosis coefficient influenced LDH levels, which ultimately influenced SaO_2_ in COVID-19 patients. These results pointed out oxidative stress status as a potential mediator in COVID-19 severity and should be considered when designing new strategies to tackle this disease. For instance, the Mediterranean diet has been proposed as a good approach to improve oxidative and inflammatory status [50]. In this line, some authors have already integrated LDH, together with NLR, D-dimer, CRP, and SaO_2_ in machine learning models in order to assess the mortality of severe COVID-19 patients [51,52,53]. Furthermore, genetic background and environmental factors should also be considered for personalized translational clinical practice [54]. The current study supports the consideration of anisocytosis as a determinant factor for future approaches, as previously suggested [26].

This research had some limitations and strengths. The fact that it was a multipurpose cohort means the collection of data and the inclusion criteria were established prior to the objectives and hypotheses being determined. In this line, because these data were collected in 2020, there were no stipulated guidelines about clinical management, which could impact the interpretation of the data. However, the huge amount of collected clinical parameters causes this database to be a good tool to study the clinical framework of COVID-19 in European ancestry. The results may not be extrapolated to other populations, such as Latin or Asian, that present different prevalence of associated diseases and lifestyles that might influence COVID-19 severity [55]. Additionally, COVID-19 presents several genetic variants that may interact differently with these antioxidants and inflammatory markers. This investigation could be complemented by exploring the potential interaction between LDH and anisocytosis coefficient considering COVID-19 variants.

## 5. Conclusions

In conclusion, the results of this investigation suggest that these two surrogate inflammatory and stress oxidative markers, such as anisocytosis and LDH, show interaction and are important determinants of COVID-19 severity measured by SaO_2_ levels. Therefore, the oxidative stress status should be considered to improve the current clinical understanding of COVID-19 and could contribute to enhance therapy prescription in a precision medicine framework.

## Figures and Tables

**Table 1:** Comparison of clinical and phenotypical variables of COVID-19 infected patients according to oxygen saturation.

Variables	O_2_ Sat ≤ 94 (*n* = 958)	O_2_ Sat > 94 (*n* = 850)	*p* Value
Phenotypical Variables			
Age (y)	70 ± 0.5	66 ± 0.6	**<0.001**
Sex (*n*, %)			
Male	605 (63.2)	479 (56.4)	**0.003**
Female	353 (36.8)	371 (43.6)	0.17
Days of hospitalization (d)	9 ± 0.2	7 ± 0.2	**<0.001**
Biochemical Variables			
Oxygen saturation (%)	88 ± 0.2	96 ± 0.04	**<0.001**
Glucose (mg/dL)	134 ± 2	120 ± 2	**<0.001**
Anisocytosis coefficient (%)	13.1 ± 0.1	12.7 ± 0.1	**0.001**
Prothrombin activity (s)	72.4 ± 0.7	74.3 ± 0.8	0.06
LDH (U/L)	682.6 ± 12.1	529.6 ± 11.2	**<0.001**
D dimer (µg/mL)	2576.7 ± 273.1	2095.5 ± 337.6	**<0.001**
AST (U/L)	42.6 ± 1.9	36.9 ± 4.1	**<0.001**
ALT (U/L)	51.5 ± 2.5	42.8 ± 5.6	**<0.001**
AST/ALT ratio	1.5 ± 0.02	1.4 ± 0.03	**0.04**
FIB—4 index	3.1 ± 0.1	2.5 ± 0.1	**0.001**
APRI index	0.7 ± 0.02	0.6 ± 0.06	0.17
C reactive protein (mg/L)	129.8 ± 3.5	67.5 ± 2.9	**<0.001**
Platelets (×10^3^/µL)	226.7 ± 3.3	217.8 ± 3.4	0.09
Creatinine (mg/dL)	1.05 ± 0.02	1.00 ± 0.02	0.12
Hemoglobin (g/dL)	14.0 ±0.06	13.7 ± 0.07	**0.02**
Eosinophils (×10^3^/µL)	0.03 ± 0.002	0.05 ± 0.01	**0.03**
Leukocytes (×10^3^/µL)	8.3 ± 0.2	6.9 ± 0.1	**<0.001**
Lymphocytes (×10^3^/µL)	1.1 ± 0.03	1.3 ± 0.07	**0.002**
Monocytes (×10^3^/µL)	0.62 ± 0.08	0.56 ± 0.01	0.52
Neutrophils (×10^3^/µL)	6.5 ± 0.1	4.9 ± 0.1	**<0.001**
NLR	8.6 ± 0.4	5.1 ± 0.2	**<0.001**
Variables of Comorbidities			
Diabetes mellitus (%)	142 (14.8)	81 (9.5)	**0.001**
Cardiovascular disease (%)	188 (19.6)	131 (15.4)	**0.02**
Overweight and obesity (%)	63 (6.5)	35 (4.1)	**0.02**
Anemia (%)	44 (4.6)	40 (4.7)	0.89
Liver diseases (%)	28 (2.9)	24 (2.8)	0.91
Gastrointestinal diseases (%)	121 (12.6)	127 (14.9)	0.14
Dementia (%)	106 (11.0)	92 (10.8)	0.89
Endocrine diseases (%)	344 (35.9)	237 (27.8)	**<0.001**
Other respiratory diseases (%)	698 (72.8)	451 (53.0)	**<0.001**
Kidney diseases (%)	121 (12.6)	85 (10.0)	0.08
Hypertension (%)	348 (36.2)	226 (26.6)	**<0.001**

Values are expressed as means ± standard errors. *p* values for quantitative variables were calculated using Student’s *t*-test or Mann–Whitney U test, according to the distribution of data. *p* values for qualitative variables were calculated using chi squared and compared groups of oxygen saturation. *p* value in bold type means significant difference <0.05. LDH: lactate dehydrogenase; AST: aspartate aminotransferase; ALT: alanine aminotransferase; APRI: AST-to-platelet ratio index; FIB-4: fibrosis-4 index; NLR: neutrophils-to-lymphocytes ratio; and CRP: C-reactive protein.

**Table 2:** Comparison of clinical and phenotypical variables of COVID-19 infected patients according to lymphocytes level.

Variables	Lymphocytes ≤ 1.05 (*n* = 982)	Lymphocytes >1.05 (*n* = 826)	*p* Value
Phenotypical Variables			
Age (y)	70 ± 0.5	65 ± 0.5	**<0.001**
Sex (*n*, %)			
Male	548 (55.8)	592 (71.7)	**0.001**
Female	434 (44.2)	234 (28.3)	**0.002**
Days of hospitalization (d)	8.9 ± 0.2	7.4 ± 0.1	**<0.001**
Oxygen saturation (%)	91 ± 0.2	93 ± 0.2	**<0.001**
Biochemical Variables			
Glucose (mg/dL)	133 ± 1.6	123± 1.3	**<0.001**
Anisocytosis coefficient (%)	13.1 ± 0.1	12.7 ± 0.1	**<0.001**
Prothrombin activity (s)	72.0 ± 0.6	75.4 ± 0.6	**0.002**
LDH (U/L)	657.1 ± 11.2	578.9 ± 12.5	**<0.001**
D dimer (µg/mL)	2504.6 ± 315.4	2311.5 ± 258.8	0.63
AST (U/L)	47.7 ± 1.3	47.2 ± 4.7	0.91
ALT (U/L)	39.9 ± 1.4	41.2 ± 3.4	0.72
AST/ALT ratio	1.5 ± 0.04	1.3 ± 0.02	**0.002**
FIB—4 index	3.2 ± 0.09	2.4 ± 0.1	**<0.001**
APRI index	0.7 ± 0.02	0.6 ± 0.05	**0.04**
C reactive protein (mg/L)	128.1 ± 3.4	76.7 ± 2.8	**<0.001**
Platelets (×10^3^/µL)	207.7 ± 2.6	241.9 ± 3.4	**<0.001**
Creatinine (mg/dL)	1.04 ± 0.02	0.99 ± 0.02	0.12
Hemoglobin (g/dL)	13.6 ± 0.06	14.0 ± 0.06	**<0.001**
Eosinophils (×10^3^/µL)	0.02 ± 0.001	0.06 ± 0.009	**<0.001**
Leukocytes (×10^3^/µL)	7.3 ± 0.1	7.9 ± 0.1	**0.005**
Lymphocytes (×10^3^/µL)	0.7 ± 0.006	1.6 ± 0.05	**<0.001**
Monocytes (×10^3^/µL)	0.4 ± 0.008	0.7 ± 0.07	**<0.001**
Neutrophils (×10^3^/µL)	6.2 ± 0.1	5.4 ± 0.1	**<0.001**
NLR	10.4 ± 0.4	3.7 ± 0.08	**<0.001**
Variables of Comorbidities			
Diabetes mellitus (%)	124 (12.6)	156 (18.9)	0.64
Cardiovascular disease (%)	191 (19.4)	210 (25.4)	**0.03**
Overweight and obesity (%)	56 (5.7)	69 (8.3)	0.67
Anemia (%)	56 (5.7)	47 (5.7)	**0.01**
Liver diseases (%)	32 (3.2)	43 (5.2)	0.95
Gastrointestinal diseases (%)	153 (15.6)	153 (18.5)	0.87
Dementia (%)	112 (11.4)	130 (15.7)	0.27
Endocrine diseases (%)	333 (33.9)	379 (45.9)	**0.01**
Other respiratory diseases (%)	646 (65.8)	739 (89.4)	**<0.001**
Kidney diseases (%)	126 (12.8)	132 (15.9)	**0.04**
Hypertension (%)	341 (34.7)	381 (46.1)	**0.04**

Values are expressed as means ± standard errors. *p* values for quantitative variables were calculated using Student’s *t*-test or Mann–Whitney U test, according to the distribution of data. *p* values for qualitative variables were calculated using chi squared and compared groups of lymphocytes. *p* value in bold type means significant difference < 0.05. LDH: lactate dehydrogenase; AST: aspartate aminotransferase; ALT: alanine aminotransferase; APRI: AST-to-platelet ratio index; FIB-4: fibrosis-4 index; NLR: neutrophils-to-lymphocytes ratio; and CRP: C-reactive protein.

**Table 3:** Comparison of clinical and phenotypical variables of COVID-19 infected patients according to D-dimer levels.

Variables	D-Dimer ≤ 743	D-Dimer > 743	*p* Value
	(*n* = 723)	(*n* = 1085)	
Phenotypical Variables			
Age (y)	64 ± 0.5	72 ± 0.5	**<0.001**
Sex (*n*, %)			
Male	456 (63.1)	417 (38.4)	0.23
Female	267 (36.9)	668 (61.6)	**0.001**
Days of hospitalization (d)	7.7 ± 0.2	8.3 ± 0.2	**0.03**
Oxygen saturation (%)	93 ± 0.2	90 ± 0.3	**<0.001**
Biochemical Variables			
Glucose (mg/dL)	123.7 ± 1.7	133± 1.9	**<0.001**
Anisocytosis coefficient (%)	12.4 ± 0.1	13.1 ± 0.1	**<0.001**
Prothrombin activity (s)	73.2 ± 0.8	73.8	0.62
LDH (U/L)	550.2 ± 7.6	712.5	**<0.001**
D dimer (µg/mL)	460 ± 6.1	4362.9 ± 395.4	**<0.001**
AST (U/L)	41.7 ± 1.2	54.9 ± 5.8	**0.02**
ALT (U/L)	39.5 ± 1.7	43.7 ± 4.1	0.34
AST/ALT ratio	1.3 ± 0.02	1.6 ± 0.05	**<0.001**
FIB—4 index	2.4 ± 0.1	3.1 ± 0.1	**<0.001**
APRI index	0.5 ± 0.02	0.7 ± 0.06	0.07
C reactive protein (mg/L)	85.6 ± 3.1	132.2± 4.3	**<0.001**
Platelets (×10^3^/µL)	218.2 ± 3.1	245.5 ± 4.1	**<0.001**
Creatinine (mg/dL)	0.9 ± 0.01	1.1 ± 0.03	<0.001
Hemoglobin (g/dL)	14.1 ± 0.06	13.5 ± 0.1	**<0.001**
Eosinophils (×10^3^/µL)	0.03 ± 0.002	0.05 ± 0.01	0.17
Leukocytes (×10^3^/µL)	7.0 ± 0.1	8.5 ± 0.2	**<0.001**
Lymphocytes (×10^3^/µL)	1.2 ± 0.03	1.2 ± 0.06	0.66
Monocytes (×10^3^/µL)	0.5 ± 0.01	0.6 ± 0.1	0.18
Neutrophils (×10^3^/µL)	5.2 ± 0.1	6.6 ±0.1	**<0.001**
NLR	5.9 ± 0.2	8.7 ± 0.6	**<0.001**
Variables of Comorbidities			
Diabetes mellitus (%)	210 (29.0)	281 (25.9)	<0.001
Cardiovascular disease (%)	103 (14.2)	158 (14.5)	**0.04**
Overweight and obesity (%)	45 (6.2)	34 (3.1)	0.23
Anemia (%)	16 (2.2)	40 (3.7)	**0.001**
Liver diseases (%)	13 (1.8)	26 (2.4)	**0.03**
Gastrointestinal diseases (%)	114 (15.7)	72 (6.6)	**0.002**
Dementia (%)	49 (6.7)	103 (9.5)	**<0.001**
Endocrine diseases (%)	224 (30.9)	241 (22.2)	0.22
Other respiratory diseases (%)	447 (61.8)	480 (44.2)	**0.02**
Kidney diseases (%)	48 (6.6)	98 (9.0)	**<0.001**
Hypertension (%)	200 (27.7)	263 (24.2)	**<0.001**

Values are expressed as means ± standard errors. *p* values for quantitative variables were calculated using Student’s *t*-test or Mann–Whitney U test, according to the distribution of data. *p* values for qualitative variables were calculated using chi squared and compared groups of D-dimer. *p* value in bold type means significant difference <0.05. LDH: lactate dehydrogenase; AST: aspartate aminotransferase; ALT: alanine aminotransferase; APRI: AST-to-platelet ratio index; FIB-4: fibrosis-4 index; NLR: neutrophils-to-lymphocytes ratio; and CRP: C-reactive protein.

**Table 4:** Comparison of clinical and phenotypical variables of COVID-19 infected patients according to inflammatory (NLR and anisocytosis) and oxidative stress (LDH).

Variables	NLR Ratio ≤ 4.5 (*n* = 973)	NLR Ratio > 4.5 (*n* = 835)	*p* Value ^a^	LDH ≤ 544 (*n* = 869)	LDH > 544 (*n* = 939)	*p* Value ^b^	Anisocytosis ≤ 12.8 (*n* = 936)	Anisocytosis > 12.8 (*n* = 872)	*p* Value ^c^
Phenotypical Variables									
Age (y)	64 ± 0.5	70 ± 0.4	**<0.001**	66 ± 0.6	68 ± 0.4	**0.002**	65 ± 0.5	70 ± 0.4	**<0.001**
Sex (*n*, %)									
Male	469 (48.2)	671 (80.4)	**<0.001**	594 (68.4)	549 (58.5)	0.21	575 (61.5)	597 (68.5)	0.26
Female	504 (51.8)	164 (19.6)	**<0.001**	275 (31.6)	390 (41.5)	0.02	361 (38.5)	275 (31.5)	
Days of hospitalization (d)	7.7 ± 0.2	8.3 ± 0.1	**0.04**	7.6 ± 0.2	8.3 ± 0.2	**0.009**	7.8 ± 0.2	8.6 ± 0.2	**0.005**
Biochemical Variables									
Oxygen saturation (%)	93 ± 0.1	90 ± 0.3	**<0.001**	94 ± 0.1	89 ± 0.3	**<0.001**	93 ± 0.2	91 ± 0.2	**<0.001**
Glucose (mg/dL)	116 ± 1.0	139 ± 1.8	**<0.001**	125 ± 1.5	130 ± 1.5	**0.02**	127 ± 2	128 ± 2	0.55
Anisocytosis coefficient (%)	12.6 ± 0.06	13.1 ± 0.07	**<0.001**	12.6 ± 0.06	13.1 ± 0.06	**<0.001**	11.3 ± 0.03	14.3 ± 0.05	**<0.001**
Prothrombin activity (s)	77.5 ± 0.6	70.2 ± 0.6	**<0.001**	76.4 ± 0.6	71.4 ± 0.6	<0.001	75.0 ± 0.6	72.5 ± 0.7	**0.006**
LDH (U/L)	540.8± 10.3	694.2 ± 12.8	**<0.001**	418.5 ± 2.6	817.7 ± 13.7	**<0.001**	573.8 ± 8.2	656.0 ± 13.3	**<0.001**
D dimer (µg/mL)	1301 ± 89	3447 ± 382	**<0.001**	1406 ± 143	3209 ± 346	**<0.001**	2039.4 ± 260.7	2651.5 ± 303.4	0.12
AST (U/L)	39.8 ± 1.3	54.7 ± 4.6	**0.002**	34.8 ± 4.3	58.5 ± 2.3	**<0.001**	43.2 ± 1.1	51.5 ± 4.6	0.09
ALT (U/L)	35.5 ± 1.2	45.2 ± 3.6	**0.008**	32.3 ± 3.3	47.6 ± 1.9	**<0.001**	38.5 ± 1.3	42.7 ± 3.4	0.25
AST/ALT ratio	1.4 ± 0.02	1.5 ± 0.04	**0.01**	1.3 ± 0.04	1.5 ± 0.02	**0.001**	1.4 ± 0.04	1.4 ± 0.02	0.39
FIB—4 index	2.6 ± 0.08	3.1 ± 0.1	**0.001**	2.3 ± 0.1	3.3 ± 0.09	**<0.001**	2.4 ± 0.06	3.2 ± 0.1	**<0.001**
APRI index	0.5 ± 0.02	0.7 ± 0.05	**0.04**	0.5 ± 0.05	0.7 ± 0.02	**<0.001**	0.5 ± 0.02	0.7 ± 0.05	**0.01**
C reactive protein (mg/L)	56.5 ± 1.8	147.3 ± 3.6	**<0.001**	65.7 ± 2.5	133.5 ± 3.4	**<0.001**	95.7 ± 3.1	108.8 ± 3.4	**0.005**
Platelets (×10^3^/µL)	214.0 ± 2.9	235.3 ± 3.2	**<0.001**	222.8 ± 3.3	226.3 ± 2.9	0.43	234.4 ± 3.2	216.4 ± 3.0	**<0.001**
Creatinine (mg/dL)	0.9 ± 0.01	1.1 ± 0.02	**<0.001**	0.9 ± 0.02	1.1 ± 0.02	**0.01**	0.9 ± 0.01	1.1 ± 0.02	**<0.001**
Hemoglobin (g/dL)	13.9 ± 0.05	13.6 ± 0.06	**<0.001**	13.7 ± 0.06	13.9 ± 0.05	0.09	14.0 ± 0.05	13.6 ± 0.06	**<0.001**
Eosinophils (×10^3^/µL)	0.05 ± 0.009	0.02 ± 0.001	**<0.001**	0.05 ± 0.009	0.02 ± 0.001	**<0.001**	0.05 ± 0.009	0.04 ± 0.002	0.31
Leukocytes (×10^3^/µL)	6.0 ± 0.1	9.2 ± 0.1	**<0.001**	7.1 ± 0.1	8.1 ± 0.1	**<0.001**	7.2 ± 0.1	8.1 ± 0.2	**<0.001**
Lymphocytes (×10^3^/µL)	1.5 ± 0.05	0.8 ± 0.01	**<0.001**	1.3 ± 0.05	1.1 ± 0.02	**<0.001**	1.2 ± 0.02	1.2 ± 0.05	0.72
Monocytes (×10^3^/µL)	0.6 ± 0.07	0.5 ± 0.01	0.51	0.6 ± 0.01	0.6 ± 0.06	0.95	0.54 ± 0.01	0.62 ± 0.07	0.27
Neutrophils (×10^3^/µL)	3.8 ± 0.05	7.8 ± 0.1	**<0.001**	5.1 ± 0.1	6.3 ± 0.1	**<0.001**	5.3 ± 0.1	6.2 ± 0.1	**<0.001**
NLR	2.8 ± 0.03	11.3 ± 0.4	**<0.001**	5.3 ± 0.2	8.4 ± 0.4	**<0.001**	6.0 ± 0.2	8.0 ± 0.4	**<0.001**
Variables of Comorbidities									
Diabetes mellitus (%)	116 (11.9)	164 (19.6)	0.51	117 (13.4)	163 (17.3)	0.22	97 (10.3)	149 (17.1)	**0.004**
Cardiovascular disease (%)	135 (13.8)	266 (31.8)	**0.01**	145 (16.7)	256 (27.2)	0.28	139 (14.8)	218 (25.0)	**<0.001**
Overweight and obesity (%)	50 (5.1)	75 (8.9)	0.44	47 (5.4)	78 (8.3)	0.82	51 (5.4)	58 (6.6)	0.79
Anemia (%)	33 (3.4)	70 (8.4)	**0.01**	38 (4.3)	65 (6.9)	0.73	17 (1.8)	69 (7.9)	**<0.001**
Liver diseases (%)	29 (2.9)	46 (5.5)	0.41	29 (3.3)	46 (4.9)	0.97	15 (1.6)	45 (5.1)	**<0.001**
Gastrointestinal diseases (%)	138 (14.2)	168 (20.1)	0.49	134 (15.4)	172 (18.3)	**0.04**	128 (13.6)	154 (17.6)	0.34
Dementia (%)	97 (9.9)	145 (17.3)	0.28	85 (9.8)	157 (16.7)	0.25	96 (10.2)	115 (13.2)	0.44
Endocrine diseases (%)	297 (30.5)	415 (49.7)	0.31	278 (31.9)	434 (46.2)	0.72	275 (29.4)	358 (41.0)	**0.005**
Other respiratory diseases (%)	570 (58.6)	815 (97.6)	**0.009**	517 (59.5)	868 (92.4)	0.14	547 (58.4)	682 (78.2)	**<0.001**
Kidney diseases (%)	87 (8.9)	171 (20.5)	**0.001**	100 (11.5)	158 (16.8)	0.93	77 (8.2)	148 (16.9)	**<0.001**
Hypertension (%)	304 (31.2)	418 (50.0)	0.43	276 (31.7)	446 (47.5)	0.84	286 (30.5)	364 (41.7)	**0.01**

Values are expressed as means ± standard errors. *p* values for quantitative variables were calculated using Student’s *t*-test or Mann–Whitney U test, according to the distribution of data. *p* values for qualitative variables were calculated using chi squared. ^a^ Comparison between groups of NRL. ^b^ Comparison between groups of LDH. ^c^ Comparison between groups of anisocytosis coefficient. *p* value in bold type means significant difference < 0.05. LDH: lactate dehydrogenase; AST: aspartate aminotransferase; ALT: alanine aminotransferase; FIB-4: fibrosis-4 index; NLR: neutrophils-to-lymphocytes ratio; and APRI: AST-to-platelet ratio index.

**Table 5:** Linear regression model involving inflammatory and oxidative stress markers as predictors of oxygen saturation in COVID-19 infected patients.

Model (Oxygen Saturation as Dependent Variable)	ß Coefficient ± Standard Error	*p* Value	R Squared Adj.
			0.26
Age (years)	−0.06 ± 0.01	**<0.001**	
Sex	0.52 ± 0.34	0.13	
Anisocytosis coefficient	−0.05 ± 0.11	0.63	
NLR	−0.05 ± 0.01	**<0.001**	
CRP (mg/L)	−0.01 ± 0.001	**<0.001**	
LDH (U/L)	2.81 ± 2.0	0.19	
Other respiratory diseases	−1.5 ± 0.34	**<0.001**	
Anisocytosis#LDH	0.41± 0.16	**0.01**	

Bold numbers indicate *p* value < 0.05. NLR: neutrophils-to-lymphocytes ratio; CRP: C reactive protein; and LDH: lactate dehydrogenase. # means the interaction term between anisocytosis and LDH in the regression model.

## Data Availability

The data presented in this study are available on request from the corresponding author.

## Supplementary Materials

The following supporting information can be downloaded at: https://www.mdpi.com/article/10.3390/antiox12020234/s1, Table S1: Approximate ranges considered standard for the clinical variables presented in this study.
