# Supplementary material for: An Interplay between Oxidative Stress (Lactate Dehydrogenase) and Inflammation (Anisocytosis) Mediates COVID-19 Severity Defined by Routine Clinical Markers
Source: Antioxidants (Basel). 2023 Jan 20;12(2):234. doi: 10.3390/antiox12020234 (PMC9951932; doi:10.3390/antiox12020234)
Supplement: Supplementary file 1 [file antioxidants-12-00234-s001.zip › antioxidants-2130400-supplementary.pdf]

Supplementary Table S1. Approximate ranges considered standard for the clinical variables presented in this study.

Variables	Normal clinical range
**Oxygen saturation (%)**	>95%
**Glucose (mg/dL)**	70-100
**Anisocytosis coefficient (%)**	11.5-15.4
**Prothrombin activity (s)**	11-13.5
**LDH (U/L)**	105-333
**D dimer (μg/mL)**	<0.5
**AST (U/L)**	8-33
**ALT (U/L)**	4-36
**AST/ALT ratio**	<1
**FIB-4 index**	<1.45
**APRI index**	<1.5
**C reactive protein (mg/L)**	<10
**Platelets (×10^3/μl)**	150-400
**Creatinine (mg/dL)**	0.6-1.3
**Hemoglobin (g/dL)**	12.1-17.2
**Eosinophils (×10^3/μl)**	<0.5
**Leukocytes (×10^3/μl)**	4.5-11
**Lymphocytes (×10^3/μl)**	1-4.8
**Monocytes (×10^3/μl)**	0.2-0.8
**Neutrophils (×10^3/μl)**	1.5-8
**NLR**	0.78-3.53
